# Selections

**Published:** 1882-01

**Authors:** 


### Selections.

### REMARKS ON THE TREATMENT OF CARIES OF THE SPINE IN CHILDHOOD, ESPECIALLY IN REFERENCE TO SAYRE'S PLASTER JACKET. By HOWARD MARSH, F.R.C.S., Assistant-Surgeon and Demonstrator of Orthopædic Surgery at St. Bartholomew's Hospital; Surgeon to the Hospital for Sick Children.

Two different methods are in vogue for the treatment of caries of the spine. The one confines the patient to the horizontal posture, with the object of relieving the column of weight, and of preventing muscular action. The other consists in the use of some apparatus with which the patient is allowed to go about as usual. The most recent development of the latter method is found in Dr. Sayre's plaster-of-Paris jacket and jury-mast. As these inventions have now been before the profession for some years, the time seems to have come for looking into the whole question, and for asking what are the principles on which such an affection as caries of the spine should be treated; what are the difficulties arising out of the conditions under which these principles have to be applied in different forms of the disease; and what is the absolute, and what the relative value of the means at our disposal for carrying them into effect.

In trying to ascertain what these main principles are, we may, I think, very properly turn to the case of inflammation of the joints in childhood. The nature of the disease is very similar in the two instances; and the functions of the spine, in bearing and transmitting weight, and in taking part in the various movements of the body, closely correspond with the functions of the large joints; of which the knee may be taken as a convenient example.

It is agreed that when the knee-joint is inflamed, it must be kept at rest; and, that rest may be secured, not only is the joint enclosed in some firm apparatus, but the patient is not allowed to bear any weight upon the limb. Either the parents are directed to have the limb maintained in the horizontal posture, or the patient is supplied with an instrument the professed purpose of which is to transmit the weight of the body to the ground, while the affected part is allowed to hang in a perfectly passive condition, with its functions as completely suspended as are those of a healthy joint during sleep. I say nothing of the efficacy of these instruments; this varies in different examples. I allude only to the fact that they are intended to place the joint at complete rest. How does the matter stand in the case of the spine? When a patient with spinal caries is supplied with a Sayre's jacket, and allowed to go about, how are the functions of the column performed? how is the weight above the seat of the disease supported, and transmitted to the pelvic arch, and how are the acts of walking, stooping, rising, lifting objects, etc., carried on? Either the patient is using his spine, or he is using his instrument instead of his spine. If he be using his spine, then the question arises, how is it that a patient may use a diseased spine, though he may not use a diseased knee-joint? while, if it be maintained that he is using his jacket instead of his spine—that the jacket becomes vicariously a part of the skeleton, and replaces the spine in respect to the bearing of weights and the execution of the various movements of the body—the proposition is one which seems to deserve close examination.

First, I will discuss the proposition, that the action of the jacket is to remove the superincumbent weight from the spine at the point of disease; or, what is the same thing, that it supports the weight of all the parts above the level at which caries exists. Let us suppose that the disease is in the sixth dorsal vertebræ, and, in order to simplify the question as far as possible, let us suppose that the patient is standing still. When the body of the sixth dorsal vertebræ is in process of destruction, the fifth tends to descend towards the seventh, with the result that the column bends forward, and that the parts within the angle are compressed. It is, therefore, required of the jacket that it shall take the weight of the upper part of the column off the lower part, and transmit it by a collateral route to the pelvic arch. If this is to be done, two conditions appear to be essential: 1. There must be an efficient basis on which the jacket may rest; 2. The jacket must have a secure hold on the part of the column which it is required to sustain. Let us first examine the base. In young adult females in whom the pelvis is broad, and much greater in circumference than the thorax, and in whom the waist is small, the jacket rests on the expanded iliac crests, and the adjacent shelving bony framework of the hips, and the basis of support is fairly ample. In adult males, however, the pelvis is much less expanded, and in circumference is often less than that of the thorax; while in children under seven (very frequent subjects of caries), while the circumference of the pelvis measures, say, twenty inches, that of the thorax is often from twenty-one to twenty-four inches. Thus, while the thorax and pelvis in young adult females form a cone with the base directed downward, in males, and quite as markedly in children, these parts form a cone the wide part of which is above. How, then, can the pelvis afford the jacket an efficient base on which to rest, while it transmits the weight of all the parts appended to the upper part of the spinal column? But more than this. In estimating the efficiency of the basis of support afforded by the pelvis, it must be remembered that the tendency of the column is to fall forward, and that therefore it is required that the purchase should be especially firm in front. In front, however, there is no bony point except the symphysis pubis. But this lies too far back, and it presents no horizontal surface looking upward on which the lower edge of the jacket can rest. Indeed, I believe no one supposes that the symphysis does, as a matter of fact, afford any support. Yet, besides the symphysis, there is nothing but the muscular wall of the abdomen, with intestines behind it—structures which, in respect to their capability of giving support to an instrument, may fairly be compared to a more or less tightly filled air-cushion. They yield and recede as soon as the pressure tells upon them. It is, I know, held by some surgeons that the purchase anteriorly is not on the abdominal wall, but on the anterior superior spines and the iliac crests. But can we concede that this is really the case? In children, these parts are so little salient, and are, so to say, so embedded in the abdominal wall, that the jacket cannot "clip" them. Even if they could be made to serve as points of support, would the skin over them bear the pressure representing the weight of the parts above the curvature? If they were thus used, should we not meet with pressure-sores over them? Though there is a great liability to pressure-sores over the spinous processes of the vertebræ, I have very rarely indeed met with them over the anterior superior spines or the crests of the ilia. Indeed, I believe these points, as a matter of fact—even when the jacket is pinched in above them, so that it has, what a child under seven has not, something approaching to a waist—afford but very slight support. This is evidently the view of Dr. Sayre, who says, at page 17 of his work, that "a detail of practical value is the application over each anterior superior spine of two or three thicknesses of folded cloth three or four inches in length. If these little pads be removed just before the plaster has completely set, such bony processes will be left free from pressure."

Thus, one of the great difficulties in the problem is, that no thoroughly efficient base for the support of the weight which the jacket is required to transmit can be obtained. But, next to a firm base to rest upon, the jacket should have a secure hold on the part of the thorax which it is called upon to support. It has been asserted that the jacket fits so closely, that it molds itself to the alternate ridges and hollows formed by the ribs and the intercostal space, and thus securely grasps the chest; and Dr. Sayre says that it is to be applied so that the ribs are held still, and so that the breathing is rendered diaphragmatic and abdominal. And, he adds, when the thorax is thus firmly secured, the anus and perinæum will rise and fall synchronously with the diaphragm, and the respiration be carried on without difficulty so long as these parts are free from pressure. "It is necessary, in some cases, that the patient should sit upon a chair, with a hole in the seat, like a close-stool, or use an inflated India-rubber ring, like the ordinary life-supporter" (p. 12).

But, surely, here is an instance in which theory and a fervid imagination have overleaped the bounds of what is either advisable, or even possible, in practice. We stand aghast when we are told that the thorax, during the period of its most rapid growth and development, is to be so tightly constricted that its movements in respiration are entirely arrested. And can we believe that the skin thus firmly compressed—so firmly that no sliding can take place between the bony framework of the thorax and the jacket—would remain free from severe injury? But, if we turn to what is observed in actual practice—nay, even if we follow the instructions which Dr. Sayre himself has elsewhere given us (p. 18), when he tells us that "the bandage should be placed smoothly round the body, and must not be drawn tight"—we cannot maintain that the jacket fits so compactly as we are told it ought to do. On the contrary, I have always found that the finger can be easily passed down between the jacket and the surface of the chest. In considering the importance of the hold which the jacket should have on the thorax, in order that it may remove weight from the point of curvature, we must remember that the work must be done, so to speak, on a fine scale. We cannot safely separate the diseased surfaces so as to establish any considerable interval between them. It is rather a question of moderating the mutual pressure of the opposed surfaces than of completely separating them; so that if our support is to be adequate, it must act within the tenth of an inch; in other words, if it yield by so much as the tenth of an inch, pressure at the seat of disease returns, and the instrument loses its effect. And the difficulty of maintaining this slight amount of separation is increased by the structure of the spine itself. If the portion above and that below the disease were two solid rods, any extension we applied would tend to separate their adjacent ends—that is, to diminish their mutual pressure; but in the spine, formed as it is of blocks, with elastic intervertebral discs, the extension used is to a great degree lost in the general mobility of the column, and cannot be made with any amount of precision to act on the point at which the disease is situated.

Thus, when we see that the amount to which we can safely separate the two segments of the spine which meet at the point of disease is not more than about the tenth of an inch; that the extending force must be conveyed through a column permitting, in its whole length, considerable mobility; that the apparatus we employ is a case so as to surround the spine itself, but the whole thorax and abdomen; that there is no adequate base from which the case can take its purchase; that, in fact, this base is usually no larger, and often much smaller, than the part for which support is required; that, in order that the functions of respiration may be carried on, the case must be sufficiently loose to allow the thorax some play beneath it,—I cannot but see the doubt that arises whether the jacket is competent to carry out the principles upon which it is supposed to act. Thus far, however, I have supposed the child to be standing still, and have discussed the action of the jacket without reference to muscular action. This part of the question, however, calls for some remark. During muscular action, the spine works, both as a whole and in all its parts, by leverage. Thus, for example, when the dorsal is extended upon the lumbar spine, it forms a lever; the lumbar spine serving as its fulcrum, while the erector spinæ is the power. Whenever a lever acts, it is pressed against its fulcrum. Therefore every movement of one part of the spine on another is attended with intervertebral pressure. Now, when a patient with spinal caries is fitted with a jacket and allowed to go about, what happens when he moves his spine—when he rises from the stooping to the upright position? If it were possible to detach the muscles at their insertion into the spine, and fix them to the jacket, the spine might remain passive, and be carried up in the apparatus; but, as it is, the spine, with the parts appended to it, is raised by muscular action, precisely as it is in health, and with the same amount of intervertebral pressure. It is the jacket, and not the spine, that is passive. The only effect of the jacket is to add to the weight which the spine is called upon to raise. Thus I do not see how it can be held that the spine is at rest, and that intervertebral pressure is prevented during muscular action by the use of the jacket; or how the condition of the spine differs from that of a diseased knee-joint, in which every muscular contraction is attended with inter-articular pressure.

In reply to the foregoing remarks, however, it may be objected that, after all, the value of a given method must be determined not so much by any *a priori* considerations as by the results which it yields in actual practice. This is true. I will, therefore, turn to this branch of the subject. In the out-patient room at the Children's Hospital, and more recently in the orthopædic department of St. Bartholomew's Hospital, I have applied the jacket to a large number of patients between the ages of one and eight or ten years, and have seen it very largely used by others. In many instances of disease in the dorsal region, the patients, while wearing the jacket, have complained of no pain (but this has seemed to prove nothing, for children often do not complain though they are entirely without apparatus), and their general health has remained good; but the deformity has, in the majority of the cases, steadily though slowly increased. Here and there I have met with cases in which recovery, with more or less deformity, has ensued; but the result has been limited, I believe, mainly to instances in which the disease after a time shows a tendency to undergo spontaneous repair, brought about, perhaps, by anchylosis between the articular processes, laminæ, and neighboring parts of the column, which, by arresting further caving-in, has relieved the bodies from pressure. In disease of the lumbar spine, also, I have on many occasions seen the deformity steadily increase, and at last a large iliac abscess form; but I cannot say I have ever seen any instance in which, even when the jacket was applied in the early stage, the arrest of the disease, and finally its cure, could be attributed to this method of treatment. In watching these cases, it has always seemed doubtful whether the jacket, instead of supporting the parts above the disease—which, let it be noted, include all the trunk and the head and upper extremities—does not simply add to the weight which the diseased column already has to bear.

As to the jury-mast for the treatment of disease in the upper part of the spine, I have not myself succeeded in rendering it useful; and, in cases under the care of other surgeons, its action has appeared clearly defective. In the cervical, as in the other regions of the spine, the scale of extension is graduated to a fraction of an inch; and an efficient action of the suspending straps is counteracted by the alteration of their tension, which results whenever the position of the head is changed. There is the same difficulty in keeping the straps tight as that which is met with in the perineal band, and which has led many surgeons to discard its use. Wherever I have examined a case in which the jury-mast was in use, I have, I believe I may say, invariably found that, after a few hours, the straps have become so loose as to produce no extension.

In venturing on an estimate of the value of the jacket, I do not forget that children are sometimes much relieved by its application. This fact must not, however, be allowed too much weight. A number of instances might be cited in which, though a method of treatment is acknowledged to be inefficient, it will yet to some extent control pain and other symptoms. The jacket often gives relief, not because it so completely removes intervertebral pressure and keeps the spine at rest that the disease is placed under the most favorable conditions for repair, but because it steadies the spine, and restrains both sudden and extensive movements either forward or laterally. The effect of the jacket is often seen when, in caries affecting one or more of the upper dorsal vertebræ is applied to the trunk below the disease, but does not extend above this level. Here, obviously, it can have no direct influence in supporting the part of the spine above the caries, and yet it nevertheless affords the patient considerable relief.

Having formed these views respecting the jacket, I am left to the conclusion that the best method at present known for the treatment of spinal caries is that by complete recumbency. This plan, if carefully carried out for the necessary time—extending, it may be freely allowed, from six to eighteen months, or even longer—will generally effect a cure; and it will also prevent the occurrence or increase of deformity. It is now well known that the means are at our disposal by which the distressing deformity that used to result in the course of hip-disease can be prevented, so that the patient recovers with a straight though it may be a shortened limb; and the lamentable distortions which now commonly ensue in the course of spinal caries can assuredly be prevented by the recumbent treatment, if it be applied in the early stages of the disease. I know it is objected that this method interferes with the general health, leads to bed-sores, and is very tedious. It is tedious no doubt; but this is a feature inherent in the nature of the disease, which, in this respect, resembles caries of the tarsus, disease of the joints in childhood, and gland-enlargements, slowly tending towards suppuration. As to bed-sores, they are never met with in children who are fairly well attended to, however long they may be kept recumbent, except in cases of extreme exhaustion and wasting. In ordinary instances of spinal disease, they may be avoided by the use of moderate care and the maintenance of cleanliness. And, as to the failure of the general health from mere confinement to the recumbent posture, this has assuredly been very greatly exaggerated. I have seen numerous instances in which children have remained robust and fat, even though they have been recumbent for as long as two or even three years. The causes of wasting and failing health are usually either pain or prolonged suppuration; and both these may be generally avoided if the recumbent treatment is adopted early and carried out thoroughly.

I believe it is very advisable to combine the use of some firm apparatus with the maintenance of recumbency. For this, the plaster jacket may sometimes be usefully employed, though the poroplastic felt cases invented by Mr. Cocking, of Plymouth, are, I think, decidedly preferable. These cases are very readily applied; they can be easily removed and remolded; they are very light, durable, comfortable, and by no means expensive. I have used them very largely for patients at the hospital, and have found them very satisfactory, both for acute disease during the period of recumbency, and also in the convalescent stage of the affection, when the patients are allowed to move about.

---

### A REPORT OF TWENTY CASES OF INJURY TO THE HEAD, WITH REMARKS. By JOHN C. SCHAPPS, M.D., of Brooklyn, N. Y.

Nothing is more characteristic of injuries to the head, as compared with those to other parts of the body, than the marked individuality usually presented. This lack of common features, while it imparts to each case a particular interest, also renders the deduction of general results from a number of them, as a basis of treatment for future ones, a matter of much uncertainty and difficulty. On the other hand, the study of a number of cases, guided by the peculiar features and the accumulation thus of a store of individual points, is to make the best use of the material at command. Every addition to this store is worthy of consideration.

The following cases, with the exception of the last, which occurred in private practice, were treated in St. Vincent's Hospital, New York, during the writer's residence as house surgeon. The report is made from notes taken at the time by Drs. W. H. Owens and J. F. Luby. The first fourteen were admitted during the service of Dr. Charles Phelps, and the succeeding five during that of Professor J. L. Little, as attending surgeons, through whose courtesy they are published.

*Case* 1. *Compound fracture of vault of skull, and incised wounds of scalp; operation; recovery.*—T. M., ice-man, æt. 28, was admitted April 7, 1880, having been struck several times on the head with an ice-axe. He was weak from hæmorrhage, but presented no evidence of intracranial lesion. There were three large incised wounds of the scalp. In one, two and one-half inches above the right ear, a raised edge of bone was discovered. This was movable, and was found to be a chip of the external table, one inch by one-half inch. Treatment: The fragment was removed and the bottom of the fissure sought by means of trephine and gouge. It was found not to penetrate the entire thickness of the skull. This wound was dressed with carbolized oil, and the others closed with sutures. An ice-bag was applied continuously for twenty-three days, in connection with the use of bromide of potassium, light diet, and laxatives when necessary. May 22, forty-five days after injury, he was discharged, cured.

*Case* 2. *Extensive fracture of vault, and probable fracture of base of skull; operation; recovery; sequelæ.*—A. M., boy, æt. 5, was admitted April 7, 1880, his mother, while carrying him, having fallen down stairs. He was in a state of coma, and suffering from shock. There was a large scalp tumor on right side of the head, the right pupil was dilated and fixed, while the left was contracted, and there was hæmorrhage from the right ear. Patient breathed without stertor. Treatment: Exploratory incision in scalp tumor revealed a fissure having its edges depressed, extending from near the vertex, downward into right temporal fossa, where it could not be followed. The rongeur was used to cut away the depressed edges. In this, as in a majority of cases, the use of the trephine was unnecessary, the removal of an edge of bone and subsequent application of the elevator accomplishing the result sought. The dura mater was exposed for a space of two and one-half by three-quarter inches, and proved to be uninjured. The edges of the bone were carefully smoothed, the wound dressed with carbolized gauze and carbolic acid, and an ice-bag applied. Stimulants were administered hypodermically, and later by the mouth, ʒ j every hour. The patient gradually rallied. For several weeks the right pupil continued dilated, and marked right hemiplegia—same side as injury was present. The administration of stimulants, iron and quinine, was necessary, bromide of potassium and the ice-bag being used to control cerebral activity. The wound granulated and healed without difficulty. There was present at any time only moderate fever. Discharged August 28, cured. Sequelæ: Shortly prior to discharge, the patient was noticed to have difficulty in expressing his thoughts, which was attributed to his youth. One month after leaving the hospital he had become remarkably weaker in intellect, the right pupil remained slightly dilated, and the hemiplegia had disappeared.

*Case* 3. *Fracture of vault and base of skull; operation; death; autopsy.*—A. C., male, æt. 27, was admitted April 10, 1880, having fallen thirty feet, striking his head upon a ship's deck. He was in a state of profound coma, having stertor, dilatation of right pupil, hæmorrhage from right ear and both nostrils, ecchymosis surrounding both eyes, and a large scalp tumor upon right side. He had had a marked convulsion prior to admission. Treatment: An exploratory incision revealed a fracture of frontal bones surrounding depressed fragments. The fissure extended into temporal fossa. The trephine and rongeur were used to remove the depressed bone and rough edges for about two inches of the length of the fissure. The dura mater was found distended, and was punctured, when a large quantity of blood escaped. There appeared no injury to the brain. The wound was dressed with carbolized oil. After the operation there was considerable twitching of both sides of body, especially the right, but no marked convulsion. Without recovering consciousness, on the following day the patient died. Autopsy showed a fissure extending from right frontal eminence into temporal fossa, and thence into the base. Both orbital plates and right middle fossa of the base were fractured. A large surface clot covered the right side of the brain.

*Case* 4. *Contused wound of forehead; meningitis; death; autopsy.*—C. M., boy, æt. 6, was admitted May 8, 1880, one week previously having been kicked in the forehead by a horse. He had suffered from headache, and for two or three days before admission had been lethargic. On admission he was semi-comatose, with very feeble pulse, rigidity of muscles of neck and back, contracted pupils, and marked fever. On the left side of the forehead was a small contused wound, nearly healed, covering a circumscribed induration of periosteum. Treatment: Stimulants and ice-bag to head. Twenty-four hours after admission, patient died. Autopsy showed well-marked subacute meningitis, extending from immediately beneath point of injury as a center.

*Case* 5. *Fracture of base and vault of skull, inferior maxilla and both radii; meningitis supervening after an interval of three weeks; death; autopsy.*—H. B., errand boy, æt. 14, was admitted May 13, 1880, having fallen fifteen feet to the floor, striking upon his face and outstretched hands. He was suffering from shock, and lay motionless, without stertor, unless disturbed, when he manifested hyperæsthesia and active delirium. The left side of the face was contused, and ecchymosis surrounded both eyes. He had slight hæmorrhage from both nostrils, none from ears, and no subconjunctival hæmorrhage. There was no paralysis, but the delirium gradually became constant and violent, requiring restraint. A careful examination revealed no local evidence of fracture of the vault. Both radii were fractured three-quarters of an inch from the distal extremities, with the characteristic silver-fork deformity. The inferior maxilla was fractured between the first and second left incisors. A fracture of the base was suspected from the above symptoms. Treatment: Bromide of potassium, ice-bag to head, cold applications to face, fourtailed bandage for fractured jaw, and splints, not including the wrist joints for fractured radii. Within twenty-four hours the delirium disappeared. The patient was now rational, and had only slight headache. This disappeared on the succeeding day, and he was then entirely free from any symptoms of cerebral lesion, so that the diagnosis of the fracture of the base was now abandoned. On the third day after admission a small swelling, like a bruise, was observed at the upper edge of the left side of the forehead. An opening was made, and a careful search with the probe revealed nothing. The patient was now allowed to get up. His pulse, respiration and temperature were normal, his appetite good, his sleep natural, and the special senses undisturbed. About two weeks after admission, a small, hard point, like a shot under the skin, was discovered adherent to the bone over the right eye. It was painless, and supposed to be old. The patient continued to enjoy good general health and entire absence of cerebral symptoms until June 5, twenty-three days after the injury. At this time, without ascertainable cause, he had headache, followed by chill, nausea, vomiting and delirium. P. 92, R. 42, T. $102\frac{3}{5}^{\circ}$. Diagnosis, acute meningitis. Treatment: Ice-bag, bromide of potassium in full doses and leeches behind ear. On the following day the above symptoms, especially the delirium, had markedly increased. The pupils became contracted, opisthotonos supervened. P. 120, R. 34, T. $105\frac{1}{5}^{\circ}$. On the next, the twenty-sixth after the injury, symptoms of compression occurred, and the patient died. Autopsy revealed extensive fracture of base and vault of skull. From the juncture of the saggittal and coronal sutures a fissure could be traced forward, in the median line to the base of the skull, thence through the cribriform plate of the ethmoid, to the left of the crista galli, and through the body of the sphenoid. About one and one-half inches above the root of the nose, a branch fissure was given off, which passed obliquely downward and to the left, to the outer side of the orbit, and thence through the orbital plate. This fracture was not discovered by the probe during life, because the opening had been made nearer the median line. The nodule noticed was found to be an exostosis upon the edge of this fissure, just above the frontal sinus. Both orbital plates were fractured in many directions. Little or no effort at union appeared. The membranes were found engorged, thickened, and covered with flakes of purulent lymph. The meningitis had been general, and had apparently originated in the left anterior fossa of the base.

*Case* 6. *Fracture of base of skull; large surface clot; death; autopsy.*—G. R., butcher, æt. 40, was admitted June 4, 1880, having fallen a distance of thirty feet to the sidewalk. He was semi-comatose and delirious, with a rapid and feeble pulse, and slight stertor. The left side of the face was much contused, and over the left eye was a contused wound. Patient soon became profoundly comatose, with all the symptoms of cerebral compression, and died in three-quarters of an hour after admission. Autopsy disclosed fracture of left orbital plate, and large surface clot within the dura mater, on the right side.

*Case* 7. *Depressed fracture of vault; operation; death; autopsy* —L. Z., female, æt. 17 months, was admitted Jnne 6, 1880, having fallen about thirty feet to the ground. She was in a state of coma, approaching collapse, with dilatation of both pupils and stertor. A large scalp tumor occupied the left side of the head. Treatment: An exploratory incision was made, which revealed a depressed fracture, extending from the anterior fontanelle forward to the left frontal eminence, and backward along the saggittal suture to the posterior fontanelle. The edges of the frontal portions were much depressed. These were removed with the rongeur. Stimulants were administered. Patient subsequently showed less marked symptoms of compression, but did not rally, and in seven hours after admission died. Autopsy showed fracture, as above, and extending backward into occipital bone. The brain showed no lesion, and death had apparently been caused by shock.

*Case* 8. *Compound fracture of vault of skull and laceration of eyeball; recovery.*—D. G., housekeeper, æt. 40, was admitted June 14, 1880, having been struck in the face with a heavy iron vessel. Her general condition was good, and there was no evidence of injury to the contents of the skull. The left eyeball was lacerated, causing a total loss of function. A contused wound over this eye communicated with an undepressed fracture into frontal sinus, and upward in the frontal bone. Treatment: Cold water dressing and rest in bed. July 17, thirty-three days after the injury, the wound had entirely closed, and the patient was discharged.

*Case* 9. *Concussion of brain and fracture of inferior maxilla; continued cerebral symptoms; recovery.*—M. P., expressman, æt. 30, was admitted June 17, 1880, having been kicked in the face by a horse. He was unconscious, but could be aroused. The pulse was feeble, pupils even and responding to light, the right being more sensitive. The right side of the face was much contused, and the inferior maxilla fractured upon the right side near the angle. Treatment: Quiet. On the following day, ice-bag and bromide of potassium. Patient did not recover his mental faculties, but continued in a state of semi-consciousness, with occasional low delirium, resembling the typhoid state. Constipation was marked, and difficult to overcome. It was finally suspected that the use of the ice-bag and bromide contributed to this condition of affairs. On July 9 these were suspended, with good effect. The delirium diminished, the patient gradually became rational, and on July 18, thirty days after the injury, was discharged, cured.

*Case* 10. *Compound fracture of vault of skull; strumous cachexia; recovery.*—J. R., shoemaker, æt. 52, was admitted June 18, 1880, having been struck on the head with a heavy drinking glass. He had angular curvature of the spine, with strumous cachexia, and very feeble constitution. Upon the face and scalp were a number of contused wounds, one, over left eye, leaping to an irregular, undepressed fracture of frontal bone, extending up to the frontal eminence. There were no symptoms of intracranial lesion. Treatment: Ice-bag, bromide of potassium and rest. Later, in consequence of the general bad condition, the administration of iron, qninia, and large amount of stimulants was found necessary. August 25 he was discharged, cured.

*Cash* 11. *Compound depressed fracture of vault of skull with laceration of membranes and brain; operation; hernia cerebri; death; autopsy.* J. J., school-boy, æt. 10, was admitted June 18, 1880, having received a heavy blow with a cart-rung.

Although suffering somewhat from concussion, he was able to talk intelligently and had no symptoms of compression of the brain. Above and exterior to the left frontal eminence was a contused wound communicating with a depressed fracture of the skull. The patient was irritable and even violent when the wound was approached. Treatment: It was necessary to administer ether, as the patient was otherwise uncontrollable. The fracture was found to be saucer-shaped, one inch in diameter, having its center depressed about one-fourth of an inch. The sharp edge of a fragment had pierced the membranes and penetrated into the brain, making a wound one-half inch long. The fragments were removed and the rough edges cut away with the rongeur. Carbolized cold water dressing and an ice-bag were applied, and bromide of potassium (gr. v, ev. 2 hrs., varied p. r. n.) administered. P. 120, R. 32, T. 104°. In a few days the brain began to protrude through the opening in the membranes. The protruded mass gradually increased in size, sloughed, and was accompanied by high fever. Light compresses were applied with a view of controlling the hernia, but their application was followed by convulsions and the attempt was abandoned. Two weeks after the injury the mass had reached the size of a hen's egg, was very fetid, and was discharging large clots of disintegrated brain tissue. Up to this time the patient had retained perfect intelligence, but was exceedingly irritable. Tonics and small quantities of stimulants were administered. The protruded mass was supposed to contain an abscess, and punctures were made into it. These were found to be perfectly painless, however deep, while the surrounding membrane was acutely sensitive. Sixteen days after admission, twitching of the face and the right upper extremity were manifested. Two days later, one or two slight convulsions, more marked on the right side, occurred. Hæmorrhage from the mass now supervened and continued until July 7, nineteen days after the injury, when the patient died. Intelligence was retained until twenty-four hours before death. Autopsy: The body was much emaciated, and all the internal organs found to be very anæmic. The membranes were adherent to the brain all over the left hemisphere. This hemisphere was almost entirely softened and disintegrated. The right hemisphere and the base appeared normal.

*Case* 12. *Compound fracture of vault of skull; recovery.*—M. M., laborer, æt. 28, was admitted June 24, 1880, having been struck on left side of head and face with a heavy carving knife. The wound communicated with an undepressed fissure extending backward from just behind the left mastoid process. He had no symptoms of intra-cranial lesion. Treatment: Cold water dressing was applied, bromide of potassium administered, with a cathartic when necessary. July 30—thirty-six days after injury, he was discharged cured.

*Case* 13. *Compound fracture of vault and base of skull with laceration of brain; death; autopsy.*—M. R., longshoreman, æt. 29, was admitted July 6, 1880. A heavy iron hook, swinging from a derrick, had torn its way through the anterior part of the skull. The patient was unconscious, and had dilatation of both pupils and a rapid and feeble pulse. He had had one convulsion before and one after admission. An irregular lacerated wound, about two inches in length, extended across the forehead and presented commingled fragments of bone and brain tissue. A triangular piece of bone an inch in diameter was deeply imbedded in the brain. There was considerable hæmorrhage from the longitudinal sinus. Treatment: The fragments of bone were removed with the fingers and forceps. Stimulants were given p. r. n. The patient did not rally, the hæmorrhage continued, considerable brain tissue came away, and on July 8, two days after the injury, the patient died. Autopsy: A little to the left of the median line of the frontal bone was an opening one by two inches. From the junction of the sagittal and coronal sutures, two fissures were found to extend; one in the median line along the edge of the above opening, thence into the base of the skull, passing through the ethmoid; the second obliquely forward and to the left to the base, where it passed into the orbital plate at its outer part. The left side of the frontal bone, thus included and containing the opening, was attached to the base by only a small portion, and was elevated about one-eighth inch. At the right parietal eminence was a depressed fracture, the inner table being much splintered. The membranes and brain at the site of each fracture were extensively lacerated.

*Case* 14. *Fracture of vault and base of skull and inferior maxilla; operation; hernia cerebri; death; autopsy.*—W. S., driver, æt. 17, was admitted July 28, 1880. He had fallen thirty feet, striking his head upon a board floor. He was semi-conscious, and manifested active delirium when disturbed. Ecchymosis surrounding both eyes was so extensive that the pupils could not be seen. He had no stertor nor paralysis. The pulse was rapid and very feeble. Over left frontal eminence was a large scalp tumor. There was a profuse hæmorrhage from both nostrils and free escape of cerebro-spinal fluid from the left ear. The inferior maxilla was fractured between the first and second left incisors, and the right middle metacarpal bone was fractured. Treatment: Stimulants were administered hypodermically and by the mouth. On the following day the patient had rallied considerably. Ether was administered because of the excessive irritability, and an exploratory incision made into the scalp tumor. A comminuted, depressed fracture was found and several large fragments of bone removed. The rough edges were cut away with the rongeur, the use of the trephine being found unnecessary. The dura mater was found to be contused and distended. It was pierced but no blood escaped at the time. The wound was dressed with lint and carbolic acid, and an ice-bag was applied. Stimulants were ordered p. r. n., and bromide of potassium in full doses. The condition of the patient—semi-conscious, except when disturbed, then violently delirious—continued, with the addition of high fever; on the third day the temperature rising to 108°. The cerebro-spinal fluid continued to escape freely from the ear, and some oozing of dark blood with slight protrusion of brain occurred from the wound. On August 1, the fourth day after the injury, he died. Autopsy: A fissure was found extending from the opening at left frontal eminence across the median line to right supra-orbital foramen. A second was traced from left supra-orbital foramen backward across anterior and middle fossæ, and through the petrous portion of the temporal bone. The body of the sphenoid, and the cribriform plate of the ethmoid were fractured, and both orbital plates comminuted. The membranes were adherent and thickened, and showed inflammatory changes. These were more marked near the opening, at which place the brain was disintegrated.

*Case* 15. *Surface-clot aud uræmia; death; autopsy.*—M. P., housekeeper, æt. 48, was admitted August 9, 1880. About twenty-four hours previous, while supposed to be intoxicated, she had fallen and struck her head against a door. She had been unconscious since that time. No history of convulsions could be obtained. On admission the patient was in a state of profound coma. She had dilatation and immobility of right pupil, and a feeble pulse of 112. Over the left side of forehead was a large scalp tumor, and over right a contused wound. There were present œdema of extremities and albuminuria. Exploration of scalp tumor and wound revealed no fracture. Treatment: Mustard was applied over the kidneys, and elaterium and jaborandi administered. In eighteen hours after admission patient died. Autopsy: A large clot was found on right side between dura mater and skull. There were signs of an old meningitis. The abdomen was not examined.

*Case* 16. *Fracture of vault and base of skull; surface-clot; death; autopsy.*—G. M., boy, æt. 4, was admitted August 26, 1880, having fallen about fifteen feet to the flagging, striking upon his head. He was in a state of profound coma, with cold extremities and feeble pulse. There were present dilatation and immobility of the left pupil, hæmorrhage from the left ear, and sero-sanguinolent discharge from the right. Ecchymosis surrounded the right eye. A large scalp tumor, through which a fracture could be felt, occupied the left side of the head. Treatment: Warm applications to extremities. Patient died in two hours after admission. Autopsy: A fissure about six inches in length was found extending from the left temporal fossa backward and inward to a point three inches above occipital protuberance. The middle fossa of the base on the right side was fractured. A large surface-clot was found within the arachnoid upon the left side.

*Case* 17. *Fracture of vault and base of skull; surface-clot; death; autopsy.*—C. C., laborer, æt. 40, was admitted September 16, 1880. No history of the manner of his injury was obtainable. He was in a state of coma, had a slow, full pulse, and stertorous respiration. The left pupil was contracted, the right dilated. He had marked twitching of right side of the body. Over left eye and behind right ear were contused wounds, while the left eye was ecchymosed. Treatment: Ice-bag was applied and perfect quiet maintained. Carbolized dressings were applied to the wounds. On the following day the patient manifested evidences of returning consciousness, but died on the second day after admission. Autopsy: A fracture was found extending from one and one-half inches above right mastoid process downward and backward nearly to the foramen magnum; also a second through right greater wing of the sphenoid. A surface-clot within the arachnoid was found upon the anterior portion of each hemisphere, that upon the right being the larger.

*Case* 18. *Fracture of base of skull; surface-clot; death; autopsy.*—E. S., housekeeper, æt. 40, was admitted September 18, 1880, having fallen forty feet upon her head. She was unconscious, the pulse was slow and feeble, both pupils were dilated and fixed, and there were present slight stertor and hæmorrhage from the mouth. External to the right frontal eminence was a large scalp tumor. There was no hæmorrhage from the ear. Patient died in twenty minutes after admission. Autopsy: A fracture was found on the right side of the base, extending from the middle fossa through the petrous portion of the temporal bone into posterior fossa; also a short fissure into the foramen magnum, and one through sella turcia. A large clot within the arachnoid occupied the right side.

*Case* 19. *Fracture of vault of skull, and laceration of base of brain; surface clot; death*; *autopsy.*—P. O., laborer, æt. 40, was admitted September 20, 1880. A brick had fallen upon his head from a height of forty feet. He was conscious, but slightly incoherent and irritable. He then had severe headache, but no other evidence of intra-cranial lesion. To the right of the vertex was a contused wound communicating with an undepressed fissure. Shortly after admission patient became violently delirous, requiring restraint. Treatment: Sulphate of morphia gr. 1-6, hypodermically. The wound was dressed with borated cotton and carbolic acid, an ice-bag applied, and bromide of potassium administered. The patient soon became more quiet. The left pupil was observed to become markedly dilated, the right remaining normal. A few hours later the left had become contracted. Four or five hours after admission the patient had become lethargic, but was violent upon being disturbed. On the following day well marked symptoms of cerebral compression, stertor, slow and full pulse, coma and dilatation of the left pupil supervened. On the second day after the injury these symptoms continued, the temperature rose to 105 2-5°, and the patient died. Autopsy: The fissure extended from the vertex downward, forward and to the right, to the center of middle fossa on the right side of the base. At this point the base of the brain was slightly lacerated, and presented evidence of local inflammation. A clot of blood, six by four inches, external to the dura mater, had caused marked flattening of the convex surface of the left side of the brain.

*Case* 20. *Compound fracture of vault; recovery; sequelæ.*—J. R., glassworker, æt. 14, was first seen November 5, 1880. Three days previous he had received a blow on the head from a brick. He became immediately unconscious, but in a short time was able to walk to his home. On the second day after the injury he had severe headache and symptoms of constitutional disturbance. On the following day, when first seen, he had had two well marked convulsions. When six years old the patient had fallen upon the back of his head. This was succeeded by occasional convulsions for six months, since which time he has had none up to the present. Upon examination he was found to have severe pain in the head at the point of injury, hebetude and intolerance of light and sound. The pupils were slightly contracted, equal and responded to light. The pulse was 112, temperature 102 2-5°. The bowels were constipated. A contused wound of the scalp was found just anterior and internal to the right parietal eminence, at the bottom of which was the juncture of three fissures of the skull. One extended downward and forward, one downward and backward, and the third inward and slightly forward. The periosteum had been removed for a small space. Treatment: The wound having been enlarged, a difference of level of half the thickness of the bone was found between the edges of the fissures. Elevation was urged, but under advice was not attempted. The wound was dressed with cold water and an ice-bag applied. Perfect quiet in darkened room, with liquid, unstimulating diet, bromide of potassium, gr. x, every two hours, and a saline cathartic were ordered. The convulsions did not recur; two days later the temperature and pulse became normal, and only occasional slight headache occurred. The bromide, in gradually reduced amount, and the ice-bag were continued for nearly six weeks. The patient was then allowed to get up. On December 22, nearly seven weeks after the injury, a piece of dead bone three-quarters by one-eighth of an inch was removed, and the wound healed without difficulty. Sequelæ: About two months later patient began to experience severe general headache, and became very susceptible to noise, and irritable. He was anæmic. Treatment: Syrup of the iodide of iron, gtt. xv, three times a day, and avoidance of exciting causes. A month later the symptoms had not perceptibly abated.

Some points of interest may be found in each of the foregoing cases. It is evident that these are, individually, of more practical importance than any deductions and inferences to be drawn from tabulating them as a whole. This would be equally true were the series many times as large. It would appear that the difference in plans of treatment advised by high authorities has had its origin in the ignoring of this very fact, and in deciding, in general, questions which can only be properly settled upon the merits of each particular case.

There is one measure, frequently neglected, which cannot be too strongly urged—namely, that all suspicious tumors of the scalp be incised, and the underlying skull carefully explored for a fracture. Should none exist, that fact is important. Should one be found, the proper treatment and probable result may then be intelligently considered. The addition of a clean scalp wound is a trifling consideration. The question of operation upon the skull frequently presents itself as a problem difficult of solution. The history of the case, the condition of the skull externally and the symptoms which denote its condition internally, may be considered the three factors which are distinct in each case, while the following well-known truths may represent the constant quantities in the solution of this problem:

1. Injuries to the head are serious not in proportion to the amount of damage suffered by the skull, but more nearly to that experienced by its contents.

2. The injury to the intra-cranial tissues may be produced by the same force, at the same time as that of the skull, as rupture of a vessel by the same force that causes a fracture.

3. The injury may be a consequence of that of the bone, as, primarily, laceration or compression of the membranes and brain by a fragment of bone; secondarily, by the intervention of inflammatory processes, as meningitis caused by an irritation from abnormal conditions of bone.

4. It may be partly concomitant with that of the bone, and partly caused by it, with or without the intervention of inflammatory action.

5. It may occur without any injury to the skull, as intra-cranial hæmorrhage or meningitis following a blow upon the head.

It is evident that any general plan of treatment must be based upon these constant factors alone, and therefore cannot be applied indiscriminately. But these factors furnish, however, some important conclusions. From the first of them follows, as a necessary consequence, an important consideration as to the gravity of operations upon the head.

For, as force is only dangerous in proportion as it is expended upon the contents of the skull, the careful removal of bone without infliction of injury upon these contents cannot be a very hazardous proceeding.

From the third and fourth it is equally evident that, since the abnormal condition of the bone is so great a factor in determining intra-cranial, and therefore, dangerous lesions, the importance of its removal can hardly be over-estimated. As an irritant, its action may be delayed, but the natural forces cannot remove it. The best they can do is to round it off and render it as innocent as may be, and this is only accomplished by the very process so important to avoid—that of inflammation. With reference to waiting, in hope that subsequent events may prove operative measures unnecessary, there is this to be considered: That should any abnormal conditions threatening the contents of the skull exist, not only is the case compromised by a prolonged subjection to the injury, but that any operative measure in proximity to inflamed meninges is quite different in its tendency from the same measure near parts in a healthy state. The possibility, further, of a recovery without operation, and subsequent difficulty from injured bone, as illustrated in cases 5 and 20, should not be ignored.

The question of resection of the skull, with or without the trephine, may, then, be called into consideration on account of the conditions of the bone itself, as above, or to relieve symptoms not caused by the bone, as to furnish an exit for blood or inflammatory products. Although too much emphasis cannot be laid upon the importance of avoiding generalities, and upon deciding each step upon the merits of the particular case under treatment, the conclusion seems a fair one that true conservatism is not that which waits to see what nature can do when hard pressed, but that which disregards the skull when its contents are to be considered, and which, when possible, removes a cause of harm before it has had time to effect any damage.

A proportion of cases where the propriety of operation is *sub judice* are those presenting a combination of injuries, as, for example, a fracture of vault and probable fracture of base. Where respect for record as an operator enters, that frequently decides the matter. But leaving this aside—as may be easily done in theory—it often becomes a choice of subtracting, or not, from the sum total of the patient's disabilities those possible to be removed. It would appear as though the fact of a case being considered "hopeless" would simplify the matter; for surely a hopeless case cannot be injured.

The only post-mortem results which indicate an operation upon the skull to have been improper, are those which show it to have been an active element in causing death which would not have been caused by the conditions calling for the operation. These results are rarely found.—*Annals of the N. Y. Surgical Society.*

### CLINICAL LECTURE ON THE ANTIPYRETIC TREATMENT OF TYPHOID FEVER BY BATHS, SPONGING THE BODY, AND THE WET SHEET, delivered at Bellevue Hospital, by AUSTIN FLINT, M.D., Professor of the Principles and Practice of Medicine and of Clinical Medicine in Bellevue Hospital Medical College, New York.

GENTLEMEN :—As introductory to the subject of this lecture, I ask to be indulged in giving some personal reminiscences. More than thirty years ago my interest was enlisted in the clinical study of the continued fevers. This was not long after the publication, in this country, of the translation by Bowditch of the great work of Louis, containing researches which established the clinical history of the typhoid fever in France, of that day, and not less of the typhoid fever of to-day in all countries. The observations by Gerhard, Shattuck, and others had recently been published, showing that typhus fever, as existing in Ireland, and as imported into this country, was a species of fever distinct from the typhoid fever studied by Louis. It was a mooted question at that time whether typhus and typhoid fever were varieties of one species of fever, or essentially different diseases. This question elicited much discussion at the annual meeting of the New York State Medical Society in 1850, and a committee was appointed, of which I was made chairman, to collect facts relating to the question. I was in this way led to study analytically, following the numerical method of Louis, all the cases of continued fever which, up to that time, I had recorded. The number of cases amounted to fifty-two. The results of the analytical study of those cases were embodied in a report. In 1851 I had collected the recorded histories of forty-eight additional cases of typhus and typhoid fever. I subjected these cases to an analytical study, and embodied the results in a second report. Again, in 1852, I studied in the same manner sixty-four cases of typhoid and typhus fever, which I had recorded since 1851, and the results were embodied in a third report. The whole number of cases analyzed thus were one hundred and sixty-four.

One advantage in studying these three collections of cases separately was, the results obtained from the collections severally could be brought into comparison with each other. The three reports were published in a volume in 1852. I may be permitted to refer to this volume for several reasons. It was my first-born of a bibliographical brood, which has since become somewhat numerous. It is not in a condition now to speak much for itself, as it has long been out of print. The edition was small, and, moreover, the volume was printed for the author, having only a nominal publisher, so that it never had a fair chance for much circulation. But it represents the employment of most of my leisure hours for a period of three years. Aside from the personal benefit derived from the studies, compensation for the labor was found in a remarkable correspondence with the results of Louis' researches as regards the clinical history of typhoid fever —a correspondence corroborating the accuracy of his researches, and going to show that the disease retains its historical characteristics in different countries and at different periods. I began the analytical studies with belief in the identity of typhus and typhoid fevers, but the studies converted me to the opposite opinion, and this opinion is now held by most, if not all, medical writers. In collecting my histories, I stumbled upon a number of cases of relapsing fever, and these were the only cases of this disease which, up to that time, had been reported in this country, except some recorded by Clymer, in 1846.

My subject to-day is the treatment of cases of typhoid fever by the antipyretic employment of cold water. I may mention the fact that, in my second report, I studied the effects of the wet-pack in five cases. By the "wet-pack" I mean enveloping the body in a wet sheet, and over it dry blankets, after the method of the so-called hydropathists of that time. This measure cannot act by the direct abstraction of heat; but it is entitled to be called an "antipyretic measure," for it is often followed by a considerable reduction of temperature. As such, it deserves more consideration than it appears to have received. Within late years I have known it to prove signally useful in many cases of febrile disease. When my reports were written, the thermometer had not come into clinical use, the intensity of fever being estimated by the subjective symptoms together with the sensation of heat communicated to the hand applied to the skin. The effect of the wet-pack, in my cases, was excellent; but I was deterred from continuing my observations by the occurrence of apoplectic coma in a case in which this measure had been employed with very marked immediate benefit. There was no ground for supposing that the treatment had anything to do with the occurrence of the coma; but, as a measure was then a novelty in medical practice, I considered that I would be held responsible for any accidents that might subsequently take place.

The following quotation from my second report expresses my ideas of the value of external refrigerating treatment at that time: "The direct effect of an increased disengagement of caloric, it is not improbable, may contribute to some of the evils of the febrile state. The most effective refrigerating measures, which possess much potency, are external applications, and these are *cold water* and *cool air*. Ablutions with cold water are usually grateful to the sensations of patients affected with fever, and abate, frequently in a striking manner, the increased heat and dryness. The simplicity of the measure causes it to be lightly esteemed by attendants, and sometimes, perhaps, by physicians. It is really an important part of the treatment of a large proportion of fever cases. The face, body and extremities may be sponged, in succession, several times a day, or as often as the heat and dryness of the surface return. A faithful, judicious nurse may occupy a considerable portion of the time with these ablutions to the advantage of the patient. Should cold water occasion uncomfortable sensations (which is rarely the case), tepid or even warm water will secure, by evaporation, part of the refrigerating effect. The evaporation will be more rapid if spirit be added to the water. Cologne, or other perfumed spirits, may be employed for this purpose. Cold water, taken into the stomach, exerts a refrigerating effect on the skin and the system at large. Patients should be allowed to drink freely. The refrigerating effect of cool air is important. This is one of the useful ends of free ventilation. To secure this end, the patient should be lightly covered, and ventilation between the bed-clothes attended to." These views, published thirty years ago, foreshadowed those which at this moment hold the most prominent place in the treatment of fever.

Since the commencement of the session (1881–82), we have treated in this hospital [Bellevue] several cases of typhoid fever. In all the cases, except some in which the temperature of the body did not rise above 103° F., the reliance has been mainly on antipyretic measures, together with those of palliation and support. Antipyretic measures have been for several years mainly relied upon in treating the cases of this disease which have been received in the medical division of the hospital with which I am connected. The number of cases treated antipyretically is not large, being only fifteen.* These cases, however, have been observed closely, and the histories recorded with much painstaking on the part of the members of the house-staff who have had the cases in charge. During the whole, or the greater part, of the course of the disease, the temperature and the pulse have been noted every hour or two of the twenty-four hours in most of the cases. I propose, in this lecture, to give the results of the analytical study of these cases. I shall not challenge your patience by reading the histories. To do this would be intolerably tedious. From the histories I shall select the facts bearing on the following points of inquiry: (1.) The different modes of employing, externally, cold water, and the general rules observed in their employment. (2.) The antipyretic effect obtained, the time required, the duration of the effect, and the repetitions of the different modes of employing cold water for the reduction of temperature. (3.) The mortality and the duration of the disease in these cases. (4.) The employment of quinia as an antipyretic remedy in these cases. (5.) The employment of alcoholics. (6.) The dietetic treatment and the medicinal remedies employed.

1. *The different modes of employing externally cold water, and the general rules observed in their employment.*—In a few cases the cold bath was employed, that is, the patients were placed in a bathing-tub, in water of a temperature of 80° F., and the temperature reduced by the introduction of ice to about 65° F. This mode may be called, for the sake of distinction, the ice bath. It was soon discontinued on account of the inconveniences attending it, and it was not resumed because the employment of

---

* It was subsequently ascertained that two additional cases had been overlooked.

other modes appeared to secure all its advantages. The other modes were the sponge bath and the wet sheet with sprinkling. The sponge bath was often found to be notably effective. To obtain the utmost efficiency of this mode, the whole body exposed to the air is sponged with cold water, and the sponging continued steadily for a considerable time. As will be seen presently, the temperature of the body may, in many instances, be satisfactorily reduced by this mode. The wet sheet, however, is more effective. In carrying out efficiently the latter, the body is wrapped in a wet sheet, and sprinkling with water from a watering-pot, repeated at intervals of a few moments. The patient need not be removed from the bed if it be protected by a sufficiently large India-rubber cloth, but the cot known as "Kibbe's cot" is to be preferred. An ordinary cot-bedstead answers, however, very well. This mode of refrigeration is vastly more convenient than the bath-tub; it can be better regulated as regards the degree of cold, the duration of its employment, etc.; it is less likely to prove hurtful, and it may be made equally efficient. In most of the cases the sponge bath and the wet sheet were employed at different times during the progress of the disease.

The general rule with regard to the employment of these modes was to resort to one of them whenever the axillary temperature exceeded 103° F., and to continue it until the temperature was reduced to at least 102°. The temperature during the continuance of the employment of cold is, of course, to be taken in either the mouth or the rectum. The symptoms, aside from the temperature, were noted, and if at any time the pulse became feeble, the respiration disturbed, or the lips livid, the measure was at once discontinued. Alcoholics were often given during the continuance of the sponging or of the wet sheet with sprinkling. It will be seen that the wet sheet, in many instances, was continued for many consecutive hours.

2. *The antipyretic effect obtained, the time required, the duration of the effect, and the repetitions of the different modes of employing cold water for the reduction of temperature.*—In order to present as succinctly as possible the facts pertaining to these points of inquiry, which are contained in the histories of the cases severally, I have arranged them in a tabular form, and they will appear in a paper which I am preparing for publication in *The Medical News*.

I have found, on comparing these tables in respect of the number of repetitions of the employment of cold during the course of the disease, that they present notable differences. The number of ice baths given in all the cases was five, of sponge baths 129, and of the applications of the wet sheet 64; the total number being 207; the number of repetitions of the sponge bath in one case not having been fully noted. Excluding the four fatal cases, the lowest number in any case was three, the sponge bath having been employed once and the wet sheet twice in this case. In the case offering the next lowest number, four sponge baths were given; the highest number was 51, the ice bath having been employed five times and the sponge bath 46 times in this case. The next highest number was 18, this number of sponge baths having been employed. Now, the question arises, did the employment of cold in any of the cases have an agency in preventing permanently a rise of temperature, which would have occurred had cold not been employed? I think we may answer this question affirmatively; but much allowance is to be made for the differences to be observed in different cases of typhoid fever in the degree of fever-heat, irrespective of any treatment. Cases are not infrequent in which the temperature never rises above 103°. If, however, the temperature for a short time rises to 104° or 105°, and, after the employment of cold three or four times only, there is no further rise to 103°, it seems fair to attribute a certain amount of agency to the antipyretic treatment in preventing a subsequent degree of fever sufficient to call for a repetition of the treatment.

Notable differences in respect of the length of time required to produce an antipyretic effect are to be observed. The effect was produced by the ice bath in 30 minutes, by the wet sheet in 25 minutes, and by the sponge bath in 20 minutes. These were the shortest periods required; the longest were 20, 11, and 10 hours. Between these extremes, the length of time in the different cases varied much. This was true not alone in different cases, but at different times in the same case. It is evident that there are no known laws regulating the length of time required to produce an antipyretic effect. No judgment can be formed beforehand as regards this point; it can only be determined by experimental observation, and the result of the antipyretic treatment on one day is not to be relied upon in judging of the probable effect on other days during the course of the disease.

The statements just made respecting the length of time required to produce an antipyretic effect by the employment of cold, apply still more strongly to the production of this effect. Looking over the figures in the column devoted to this heading, it is found that variations were great. For example, in one case the consecutive durations are ten days, one hour, 48 hours, and 24 hours. In another case consecutive durations are two hours and a-half, 13, three, 20, and five hours. In another case, after consecutive durations of six, 15 and eight hours, the temperature before the last of these periods being 104°, there was no future rise to 103°. There is no approach to such a regularity in the successive periods during which the antipyretic effect continues as to point to any appreciable law. After the reduction of temperature has been effected, the previous experience, even in the same case, furnishe little ground for predicting the length of time during which the temperature will remain reduced. Nor is it practicable to judge beforehand, from either the duration of the antipyretic effect or its degree, of the amount of the rise of temperature which wil take place. Nor, again, can any inference be drawn from the temperature which exists when cold is employed, whether or not hyperpyrexia will again occur.

In several instances a decline of temperature takes place after the discontinuance of the sponge bath or the wet sheet. The time during which the decline took place varied from 15 minutes to 15 hours. This fact was noted three times in one case, twice in two cases, and four times in one case. In one case the temperature was increased while the patient was in the wet sheet, reduction afterward taking place. So far as an inference is to be drawn from this collection of cases, it is not the rule, but the exception to the rule, for the temperature to decline after the discontinuance of either the sponge bath or the wet sheet.

It is not to be denied that the facts in these cases are not sufficient to test the relative value of the wet sheet and the sponge ath as contrasted with the ice bath. The latter was employe only five times and in only two cases. In each instance the tem perature was promptly reduced. But there were instances in which the wet sheet and the sponge bath proved equally effective. The facts contained in the tables show that by means of the sponge bath a satisfactory antipyretic effect may be often obtained. This being the simplest mode and most easily employed, it may be first tried, and, if it fail, the wet sheet substituted. These two modes, singly or successively employed, will, as I believe, render unnecessary the use of the bath-tub. The wet sheet may be be made more or less efficient, according to circumstances, by sprinkling at longer or shorter intervals and by using water of different temperatures.

Finally, I would call attention to the fact that in no instance could any immediate harm be attributed to either of the three modes of the employment of cold. In no instance were there symptoms following their employment so closely as to indicate that the patient had suffered injury therefrom. As a rule, the reduction of temperature was accompanied by improvement in other symptoms. Patients sometimes complained of discomfort, but this probably arose from the reluctance to be disturbed rather than from any unpleasant effects attributable to the cold; oftener they expressed a sense of comfort during its employment.

The number of cases in this collection is not sufficient for testing the influence of the antipyretic measures of treatment on the fatality of typhoid fever. In this regard, however, they may be considered as having some value. Of the fifteen cases, death took place in four. Two cases of recovery, in which the same measures of treatment were employed, were overlooked prior to the completion of the analytical study of the fifteen cases. Adding the two additional cases, the fatality was four in seventeen cases. This fatality is not far from the average rate in collections of cases otherwise treated. So far as any deduction is warrantable, it may be said that the antipyretic measures neither increased nor diminished the fatality.

The duration of the disease in the cases ending in recovery was determinable with precision in nine cases. The shortest duration was nine and the longest twenty-seven days. The mean duration was seventeen and a half days. The latter is one day and a half longer than the mean duration in forty-two cases analyzed by me thirty years ago. The commencement of the disease in the cases now and in those formerly analyzed was dated from the time of taking to the bed. In the cases now analyzed the fever was considered as having ended when the temperature had fallen to within the normal range, that is, from 98° to 99°.

It cannot be said that the mean duration in the cases now analyzed is evidence that the disease was shortened in its course by the antipyretic measures of treatment.

In the four fatal cases the shortest duration was fourteen and the longest thirty-four days. The mean duration was twenty-one and a quarter days. Post-mortem examinations were made in three of the four cases. In none of the three cases were grave complications found after death. Death took place in all by asthenia. In no case was there any ground for the belief that the antipyretic measures of treatment had any agency in the fatal termination.

3. *The use of Quinia as an Antipyretic Remedy.*—The sulphate of quinia was given in several instances, as an antipyretic remedy, in conjunction with the employment of cold. The instances are noted in the tables. How much effect may have been produced by the quinia in these instances cannot be determined with accuracy. Inasmuch, however, as the reduction of heat effected by the two was not greater or more rapid than often by cold alone, it is fair to conclude that the agency of the quinia was not great. In a few instances quinia was given for an antipyretic effect without being conjoined with cold. In one of these instances the temperature fell in eight hours from 103¼° to 102° after 30 grains of the sulphate of quinia. In another instance 20 grains were given, and two hours afterward 25 grains. In two hours after the last dose the temperature had fallen from 105° to 103¼°. In another instance 15 grains of the sulphate of quinia were given, the temperature being 104°. In five hours the temperature fell to 103¾°. Ten grains more were given, and in two hours the temperature was 102°. Ten grains more were given, and in two hours the temperature was 102¼°. A fourth dose of 10 grains was then given, and in four hours the temperature was 100½°. Ten grains three times were given on the following day, the temperature ranging from 100½° to 99°. These doses were continued during the next day, and the temperature rose to 102°. The patient was much cinchonized, and the remedy was discontinued. In another instance 20 grains of the sulphate of quinia were given, the temperature being 102¼°. In three hours the temperature had risen to 104°, and the wet sheet was then employed.

4. *The use of Alcoholics.*—In the treatment of these cases alcoholics were given to meet indications relating to the circulation. In some of the cases no alcoholics were given. The quantity given was regulated by the frequency and fullness of the pulse, and diminished intensity of the first sound of the heart as heard over the apex. Whisky was the form generally used.

5. *The Dietetic Treatment.*—The chief article of diet during the course of the disease was milk. From one quart to two quarts were given daily, lime water being added.

6. *The Medicinal Treatment, exclusive of Quinia.*—In some of the cases no drugs whatever were given. Those which were given, exclusive of quinia, had reference to the palliation of certain symptoms. Digitalis was given in some instances for its tonic effect upon the heart, and ammonia as a cardiac stimulant. Opium in small doses was sometimes given for diarrhœa. The medication was chiefly limited to these remedies:

From the study of these cases it may be concluded:

1. That by the employment of cold water externally in cases of typhoid fever, the temperature of the body may, after a variable time of the continuance of the employment, be reduced to 102° or lower.

2. After a period varying very much in different cases, and, also, at different times in the same case, the temperature, as a rule, again rises as high as, or higher than, before the reduction.

3. Repeating the employment of cold as often as the axillary temperature exceeds 103°, the number of repetitions required in different cases is extremely variable.

4. The sponge bath and the wet sheet with sprinkling may be employed to the exclusion of the bath-tub in the antipyretic treatment in cases of typhoid fever as well as of other febrile diseases.

5. These modes of employing cold water may be continued sufficiently long for the reduction of temperature to 102° or lower, and repeated as often as may be required, without risk of any immediate injury, and the study of these cases furnishes no ground for supposing that a liability to complications or accidents is thereby increased.

6. Reduction of temperature by these modes as often as it rises, in the axilla, above 103°, improves the condition of the patient. The cases now studied do not afford proof either that the fatality of typhoid fever, or that its duration is thereby diminished. The study of these cases, however, renders it possible that this proof would be afforded by a larger collection of cases.

During the period that the cases now studied were treated, seven hospital cases were recorded in which antipyretic treatment was not employed. In most of these cases the temperature did not rise above 103°, and it was for this reason that the treatment was not employed. Of these seven cases three were fatal, but I need not say that it would be unfair to draw any deduction from the contrast as regards the proportionate number of fatal cases. It is well known that, in general, resistance, toleration, and recuperation are not as well exemplified within as outside of hospitals. Moreover, in cases of typhoid fever, patients are not admitted into hospital until some days after the commencement of the disease. The clinical test of therapeutical measures, as far as fatality is concerned, is therefore best afforded by the study of cases in private practice.

7. The results of the analysis of these cases, although not sustaining the statements of Leibermeister and others respecting the controlling influence of the employment of cold externally in cases of typhoid fever, yet not only show this method of antipyretic treatment to be safe, but afford encouragement to employ it with the expectation of diminishing the severity of the disease and its danger to life.—*Medical News.*

---

*For Uremic Headache, with Deficient Renal Action:*

| | | | |
|---|---|---|---|
| ℞ | Potass. citrat.................... | ℈j; | 1.33 gm. |
| | Infus. digitalis.............. | āā ℥ss; | 15.00 fl. gm. |
| | Infus. buchu................ | | |

M. Sig.—This amount three times a day.

### A PECULIAR CONDITION OF THE CERVIX UTERI WHICH IS FOUND IN CERTAIN CASES OF DYSTOCIA.* By ALFRED HOSMER, M.D.

That extraordinary form of dystocia to which attention was directed in former communications† still retains much of the character of an interesting novelty; its importance, which is not unlikely to be underrated for the very reason that its occurrence is supposed to be rare, is not as yet generally appreciated. No allusion is made to it in the last edition of Leishman's Midwifery, published scarcely a twelvemonth since, and revised by the hand of the author. Nor is it mentioned in the second English edition of Playfair's Midwifery. Under date of June 18, 1878, Dr. Playfair writes to me as follows: "I have never seen, or at least recognized, a case of the kind; but I can quite understand the occurrence." However, in the second American edition of this writer's works, Dr. Harris has introduced the subject under the head of "tetanoid falciform constriction of the uterus."‡ In many respects this designation is suggestive and significant, yet it lacks precision, intended as it is to provide for a certain mental reservation with which a topic new to text-books is discussed by the editor, who, at the time of writing, has not been convinced that the internal os is the seat of the anomalous constriction. The title under which we first introduced this subject had its origin in some early impressions which were received in connection with the imperfect investigation of a single case. I was not ready to abandon it until the testimony of clinical observation and anatomical study should have proved what relation exists between a preternatural elongation of the cervix and that strange tetanic contraction limited to a narrow segment of circular fibers of the uterine muscle; and to what extent the amount of this elongation would indicate the degree of dystocia to be encountered, and measure the danger to which the patient is to be exposed.

---

* Read at the meeting of the Obstetrical Society of Boston, February 12, 1881.
For the sake of uniformity the original title is still used, though the condition under consideration would more properly be designed as *tonic spasm of the internal os.*

† *Journal*, vol. xcviii., No. 12, p. 360, and No. 22, p. 683.

‡ Playfair's System of Midwifery, second American Edition, page 350.

In the third American edition of this book Dr. Playfair respectfully admits the existence of this odd deformity of the uterus, and says: "I have no personal experience of this complication, which, fortunately, must be very rare." He does not attempt to treat or discuss a subject a knowledge of which he would be forced to derive mainly from sources which are neither indorsed by personal acquaintance nor certified by high professional reputation. Yet English writers, whose books teach classes of the largest possible size, must soon appreciate a new form and phrase of difficult labor, and make it a visible, if not conspicuous, object in the obstetric field; otherwise their instructions in midwifery will be incomplete, and fail to provide for one of the most serious emergencies of practice.

It is not to supposed that this Society will demand any additional proof of the possible existence of an *ante-partum* hour-glass contraction of the uterus. But there is a great diversity in cases which, for convenience and simplicity of classification, are designated by the same name, and the symptoms found in a single instance can rarely, if ever, suffice for a complete description of any morbid condition. I have therefore thought proper to place before you all the clinical material not included in my original collection which I have been able to obtain from the periodical literature of medicine. The several cases will be presented, as far as possible, in the chronological order of their occurrence.

In the *Medical and Surgical Reporter* of February 5, 1876, may be found the report of a case of hour-glass contraction The patient was a married primipara aged thirty-one. Labor began in the afternoon of April 17, 1874,* upon what was, by calculation, the one hundred and sixtieth day of pregnancy. At the end of sixty hours a dead child was delivered, and without assistance, so far as the record enables us to judge. The womb not diminishing in size, its cavity was explored for the purpose of ascertaining if it contained another child. "The placenta was found attached at the fundus † of the uterus, and it was still quite impossible to make out the kind or condition of the body above."

---

* My first and only observation was made in September, 1876.

† This statement may fairly be questioned. Those portions of the report which refer to the placentæ are somewhat confused.

Twenty hours later a consultation was held, and upon removing a placenta there was found an hour-glass contraction, the degree of which was such that there was left an opening of only one inch in diameter, through which a second fœtus could be felt. It was a slow process by which the operator reached and brought down a foot. Progress was slow, and only made by dint of persistent traction, and continued so until the constriction came to encircle the fœtal neck. At this point all movement "seemed to be entirely arrested, when, as if something was giving way, the child made rapid advances by the woman's natural efforts, and was born headless." There were two separate placentæ, both of which were duly expelled. "No flowing followed; patient slept some from exhaustion, and with but little tympanites." "On the morning of April 22 the head was found, brought nearly down by the organ's own power." The amount of hæmorrhage was not dangerous, yet the woman died on the afternoon of the eighth day, April 24. This case occurred in the practice of Dr. M. D. Goodyear, of Gorton, N. Y. It is to be regretted that his report is not made with more of fullness and detail. Such as it is, I offer it without comment.

In the *Pacific Medical and Surgical Journal*, January, 1879, Dr. W. A. Briggs, of Sacramento, Cal., relates the following facts: "A married woman, aged thirty-one, whose four previous pregnancies had terminated normally, fell in labor, at term, August 3, 1876. A few hours later the right arm of the fœtus was found to be protruding through the os. At six A.M. on the 6th Dr. B. was called in consultation. He found the patient in active labor, with no untoward symptoms and with "pelvic dimensions normal." He proceeded to turn, but upon the first search was unable to find the feet, which, together with the head, proved to be beyond easy reach, inclosed as they were in the superior uterine chamber. During a second attempt the hand came in contact with what was taken to be the fundus of the uterus, and very soon the cessation of a pain allowed the finger to pass beside the fœtal neck "through an opening in the presumed uterine roof," and for an instant the alarming impression was conveyed that the peritoneal cavity had been entered. It is soon discovered that the edge of the supposed aperture is perfectly smooth, and to the touch suggests "that a silken cord has been run beneath the mucous membrane of the womb, and tightened about the child." During a pain "the 'cord' becomes more rigid, grasps the neck of the child with my finger and almost cuts them. Such are its wonderful power and tenuity." At the instant of uterine contraction the constriction embraced the fœtus with such firmness that there seemed to be not simply contact but continuity of tissues belonging to different individuals, so that all trace of an opening disappeared. The patient was now etherized. At first the constriction was found to be as firm as ever, and the child was "held as by a vice." Force was required, yet by digital and manual dilatation the stricture was gradually overcome, and at the end of thirty minutes the hand was fairly within "the upper cavity." The passage of the fœtal head having been provided for by securing a still farther relaxation of the annular contraction, the feet were seized and brought down, and delivery was accomplished without difficulty. The child was dead. The placenta followed naturally. The patient made a slow but complete recovery. To the report of this case the writer appends some thoughtful remarks, interesting to read, but not sufficiently brief for introduction here. He insists upon that difference of anatomical structure which makes the contrast between the uterine body and the cervix, and clearly points out the relations in which these two portions of the organ respectively stand to the parturient function. He states at length, and illustrates by diagram, his theory of the manner in which the innate forces of the womb lose their natural equilibrium, and thus produce deformity and dystocia.

In the *North Carolina Medical Journal*, March, 1878, A. H. Goelet, M.D., of New York, furnishes a very meagre and unsatisfactory account of a case of ante-partum hour-glass contraction, which came to his notice July 24, 1877. The patient was a married woman, and this, her third labor, was premature, occurring in the middle of the seventh month. In due time "the uterus was discovered to be contracted in the center, the fœtus above the constriction and a finger projecting through." On account of a hæmorrhage, which in amount was sufficient to compromise the safety of the woman, it was decided to deliver at once. "With great difficulty one finger was forced through the constriction, then another, and after considerable search a foot was found." The constriction opposed the passage successively of the breech, the shoulders, and the head, so that at each of these points there came a requisition for "all the force that could be exerted with both hands." The removal of the placenta was effected with considerable difficulty. Beyond this point the record does not go. But if the foregoing report is based upon observation that is accurate and authentic, the case is one of unusual interest and significance. For it would prove that the development of an ante-partum hour-glass contraction does not necessarily depend upon those influences which may be traced to the relations existing between either the maternal pelvis or the uterine cervix and some portion of the fœtus.

Among the papers announced to be read before the Gynæcological section of the American Medical Association, at the meeting held in Buffalo, in June, 1878, was one entitled "Hour-glass Contraction of the Uterus prior to the Expulsion of the Child, by T. A. Reamy, M.D., of Cincinnati, Ohio." What I take to be this paper is reprinted under date of May 10, 1879, in the *Cincinnati Lancet and Clinic.* It contains the report of three cases, one of them being referred to in the *London Medical Gazette*, July, 1848, from *Schmidt's Jahrbücher*, vol. lxiv., p. 37, 1849. A foot-note alludes to a fourth case, "recently reported in the *American Journal of Medical Sciences.*" The paper contains some facts that are extremely interesting. It proves that the patient in whom this formidable complication occurs for the first time is not necessarily a primipara, but that it may present itself in a woman who has passed through as many as seven normal labors. It also shows how the abdominal walls may be moulded to the shape of the deformed uterus, and how readily external palpation may often recognize the constriction. As the result of careful clinical observation, it certifies to the marked differences in the physiological condition and action of the two portions of the uterus which are respectively above and below the stricture. The supposed obliquity of the constriction in one instance, raises the question of the possibility of an unequal elongation of the opposite sides of the cervical cylinder. Case II. of this series forms a very valuable acquisition to the history of this subject, illustrating, as it does, the relation which may exist between tonic spasm of the internal os and the several stages of a mutiple birth. According to the record, as the result of an eighth pregnancy, two living male children were born by a brief and easy process. They weighed, the first five and one half, the second five pounds. The uterus was found to contain a third child, and through the abdomen it was "easy to trace the rigid hour-glass contraction." "By persistent traction the hips passed through the constriction, and a dead female child of four and three quarter pounds was delivered." "Just below the umbilicus, at a point seized by the constriction, the body of the child was nearly cut in two." The placenta, with three cords adhering above the constriction, was reached and removed with the greatest difficulty, and only by the successive efforts of three physicians, each of whom experienced in a painful degree the paralyzing and disabling effect of the severe pressure to which the hand was subjected in its vigorous and forcible efforts to overcome the occlusion, and obtain possession of the true uterine cavity. The woman speedily died.

The *Boston Medical and Surgical Journal*, in its issue of May 27, 1880, published the proceedings of the Society, at the regular meeting held in October, 1879. Therein is included the report of a case made by Dr. L. R. Stone, in which a firm constriction, encircling the fœtal neck, seriously retarded the progress of labor, rendered instrumental interference necessary, and in some way determined a still birth. The mother recovered. It is worthy of note that a degree of relaxation in the stricture seemed to be produced by the use of ether.

On page 397 of the *British Obstetrical Journal*, September, 1879, may be found "Note of a case of spasmodic contraction of the lower uterine segment during the first stage of labor." It was under this title that Dr. Angus MacDonald made a communication to the Obstetrical Society of Edinburgh, at a meeting held February 12, 1879.* Mrs. C., a chronic epileptic, then advanced ten or twelve hours in her fifth labor, was visited in consultation by Dr. MacDonald at 9.30 P.M., December 16, 1875. Pains

* The Obstetrical Society of Boston had discussed this subject as early as February 9, 1878.

were severe. "Immediately above the cervical segment, which was half dilated and easily dilatable, the lower uterine segment was felt to project or bulge in a peculiarly rigid or shelving manner," and to be as "hard as a deal board." Such was the rigidity of the parts that the presentation could not be ascertained. "The perinæal and levator ani muscles, as well as the lower uterine segment, were observed to be thrown into a condition of intense spasms by every attempt to perform a vaginal examination." The full impression of chloroform removed the spasm at once, and through the unbroken membranes a small head was found to be presenting at the time. During each pain the external os and cervical canal remained flaccid, but the inner os was closely shut, and the head, instead of being depressed by uterine action, was elevated very distinctly. In due time the need of artificial aid was obvious, and with the assistance of chloroform anæsthesia, version was performed with ease, and the child was saved. "The placenta was expelled spontaneously about fifteen minutes after the birth of the child." The record contains no intimation that the maternal convalescence was not a rapid and prosperous one. The reporter alludes to a possible complication of version, which is sometimes fatal to the child, and which consists "of the trouble that is occasionally experienced in getting the after-coming head through the spasmodically contracted lower uterine segment."

The species of case which gives title to his communication, is in his experience unique. The conclusion must then be that it is only in an extremely mild and manageable form that he has witnessed ante-partum hour-glass contraction.

With one exception, the ideas which found expression in the discussion that followed were based upon the theory which located the spasm in the internal os and adjacent muscular tissue of the uterine body, and acquitted the cervix of all participation in the abnormal and perverted condition. The "why" of this thing did not escape attention, and the question receives a provisional answer, as follows: "It would, therefore, appear to me that the predisposing cause in this case was a neurosis affecting the vagina and the lower part of the uterine body, but not extending to the upper ranges of the body, or to the fundus uteri, and which seemed to act in such a manner, on slight stimulus, as to throw the parts within its reach into a condition of spasmodic contraction. The pressure of the head against the internal os during a pain would appear of itself to have been sufficient to start this spasmodic condition, or, in other words, to have probably acted as the proximate cause of the spasmodic condition." Science may gain something if future observers will bear in mind the hint here given as to the relation existing between individual temperament and tonic contraction of the internal os. In my first paper I distinctly stated the possible aggravation of such contraction through the reflex action of measures that were intended to be remedial.

In view of the terms of the title under which I now for the third time ask the attention of the society to a dangerous and anomalous condition, which in November, 1876, was introduced under the name of ante-partum hour-glass contraction, I desire to quote Dr. J. Matthews Duncan. His article On Two Contrasted Forms of Weak Labor, copied from the *Obstetrical Journal*, February, 1878, page 705, may be found in Braithwaite's Retrospect, Part 67, July, 1878, page 184. In one case labor is weak because the uterus of the elderly multipara is feeble and inert, endowed with only a small remainder of its original energy, and incapable of vigorous and efficient action. In the other case, the subject is generally a primipara, or a young woman having a special nervous mobility. The labor is practically weak, not through any deficiency in the inherent muscular power of the womb, but for the reason that the contractile force of the organ, instead of undergoing the normal conversion into the propulsion and expulsion of the child, is expended in the production of deformity. In other words, the body of the womb acquires a permanent retraction; with each succeeding pain becomes a little shorter in its longitudinal dimension, always adding something to the thickness and strength of its wall; while the cervix is elongated and attenuated in a corresponding degree; the fœtus becomes stationary, and the condition of labor loses its parturient character. The disproportion between the two portions of the womb may be so great that the lower margin of the uterine body is sometimes raised almost to the level of the umbilicus. In both these forms of weak labor, "the child is brought into the world by a very small expenditure of force." Dr. Duncan cites the case of an "excessively nervous" woman whose three labors were all, in a similar way, busy and painful, but tedious. "When the head reached the perinæum, while the pains continued severe, progress was arrested." It was only at the third confinement that the nature of the case was exactly ascertained. At that time "delivery by forceps was effected without any effort worthy of the name of pulling, the child was little more than lifted out of the passages."

Without forgetting the allowance that is due to irregularity in pelvic size or shape, let us make a comparison, placing upon one side that case in which the uterus, by the retraction of its body and the elongation of its neck, loses the relation and proportion that naturally exist between the two portions which in their combination constitute the organ; and in which, although spontaneous delivery is extremely improbable, the removal of the child is effected by artificial means with surprising facility and with absolute safety. Upon the other side, we will place the case in which uterine deformity of the most aggravated kind is produced by the introduction of a third element; by the addition of a tetanic spasm of the whole circle of the internal os to the retracted body and elongated cervix, and in which not only is spontaneous delivery simply impossible, but the termination of labor is reached with a difficulty that is beyond all description, and can be appreciated by those alone who have met and overcome it, and have been eye-witnesses of the appalling danger to which it exposes both the lives that are involved in every case of child-birth, and which natural labor was never intended to put in jeopardy.

Trite and old is the saying that new things are not to be found on this side of the sun. Possibly the older obstetricians failed to appreciate all that passed before their eyes or came in contact with their hands, just as the old physicians accepted identity when there existed a similarity only the most remote, and in diagnosis overlooked distinctions which now irresistibly force themselves upon the attention. But within seven years, that is, since April, 1874, by a coincidence that is not without precedent in the history of even important discoveries, ante-partum hour-glass contraction has been observed in so large a number of cases as to modify the impression of extreme rarity and extraordinary character which was conveyed by the first report of a case made to this society. The wide geographical distribution of the instances here collected proves that this particular complication of labor does not take its origin in accident or influence of place, but that it forms a legitimate and indisputable part of universal midwifery, and is entitled to a high rank among those difficulties and obstacles to delivery which make the process of child-bearing in any degree unnatural. Different observers, in entire ignorance and independence of each other, have made their several descriptions in words and phrases so nearly identical that there can be no doubt that they have all been dealing with the same thing.

I will not attempt a careful analysis of the several cases which constitute the substance of this communication, but upon making a general review of them, I would draw the following conclusions, and ask their provisional acceptance:

1. Simple elongation of the cervix, even though it be excessive, disables the uterus by perversion of its force, renders spontaneous expulsion improbable, but in connection with artificial delivery does not produce a condition of things to which the term dystocia can be applied with any propriety or significance.

2. Tonic spasm of the internal os may in single labor be developed so early as to imprison the whole fœtus in the cavity of the uterine body, and in a multiple labor its production may be so postponed as to interfere only with the birth of the last child. Its existence cannot necessarily be referred either to pelvic deformity, to extreme elongation of the cervix, nor to the occupation of the cervical cavity by any portion of the unborn child.

Finally, I would call attention to the facts that tonic spasm of the internal os has shown a marked tendency to recur in the successive labors of those who have once or even twice survived the danger to which it exposed them, and that the patient in whom it occurs for the first time is not necessarily a primipara.

The form of difficult labor to which this article refers has sometimes been spoken of as "Bandl's dystocia." That obstetrician published in 1875 Rupture of the Uterus, and in 1876 Relation of the Uterus and Cervix in Pregnancy and during Labor. But I am not aware that in either publication he describes tonic spasm of the internal os. A competent medical friend, recently a student in Vienna, and thoroughly familiar with the German language, having searched at my request, writes under date of February 24, 1881: "I have looked carefully through the two pamphlets, and find no mention at all of a spasmodic contraction of the internal os."—*Boston Med. and Surg. Journal.*

---

ON THE TREATMENT OF THE VARIOUS FORMS OF CONSUMPTION. BY ROBERTS BARTHOLOW, M.D., LL.D., Professor of Materia Medica and Therapeutics in the Jefferson Medical College. Philadelphia. (Concluded from page 669, December, 1881.)

I will now pass to the consideration of particular forms of phthisis.

You have seen recently several cases of caseous phthisis, in which there were considerable areas of infiltration; the period of inflammation had passed, and the products accumulated in the alveoli of the lungs were undergoing casceation. Now the problem for solution in these cases is, how shall we obtain the softening and extrusion of the caseous material without destruction of the pulmonary tissues? Although it is generally held that we possess no direct means of acting on these caseous deposits, and that they can be removed only by the improvement which we may effect in the bodily condition, I have reason to believe that we can reach and act on them to a considerable extent. The salts of ammonia have the power to increase the alkalinity of the blood, to prevent coagulation of fibrin, and to dissolve the albuminous exudations. Whilst the remedies were administered I have repeatedly observed the absorption and disappearance of caseous deposits. It is obvious the smaller in extent and the more recent, the more amenable to the action of the remedy. Probably the best mode of administration of the ammonia is to dissolve the carbonate in solution of the acetate (gr. v-x-℥ ss), or when it is desirable to give the iodide with the carbonate, they may be administered in an emulsion together. The duration of the administration will be governed by the effect on the caseous deposits and by the forbearance of the digestive organs, but the remedies must be used for many months in most cases. By giving them properly diluted before meals, and allowing the stomach periods of rest, they may be continued for lengthened periods without ill results.

The range of temperature, and the amount and kind of interference required, are very important subjects. With regard to the character of the fever it must suffice for me to insist upon the fact, which I have often stated, that the febrile movement of the stage of exudation and deposit is different in character from that of the period of softening and extrusion. The former is inflammatory; the latter septicæmic. During the inflammatory stage we are concerned to employ means to lesson exudation, and to favor its immediate liquefaction and discharge. These means are quinia, digitalis, aconite, jaborandi, etc. When, however, the alveoli are blocked with the caseous deposits our attention must needs be directed to secure a favorable disposition of this material. When the caseation is completed there is a temporary subsidence of the active symptoms, which may continue for some time; the fever abates notably or disappears; the general state correspondingly improves; the cough is less troublesome; the expectoration slight; and there is, in fact, a delusive appearance of returning health. The scene changes when the process of softening begins, and moist sounds are audible in abundance where bronchial voice and breath were heard a short time before. Now there is an extensive area of pus formation, and the fever which attends it is a septicæmic fever. Observe the attendant phenomena; there is a daily sensation of coldness, of chilliness, or a distinct chill, some time during the morning, and for a short time before it the temperature had been at normal or even below; then a fever comes on, the mercury rising to 103°, 104°F.; and the whole process terminating in a profuse sweat. In respect to this fever the practical point I wish to impress on you is, that the best means of keeping down the temperature are rest, and the administration of remedies to restrain the sweats, and to quiet the cough. By rest I mean a condition of repose without exercise, except such muscular movements as are required in dressing, in walking from one room to another on the same floor, etc. There are sound physiological reasons for this recommendation. Rest lessens the consumption of material, the wear of tissue, and the production of heat, and conversely exercise increases the consumption of material, wastes the tissues, and increases the production of heat. Applying the physiological data to the subject before us, I must admit that exercise is theoretically prejudicial to the phthisical suffering from septicæmic fever. I have made a number of careful observations with a standard thermometer to ascertain the effect of exercise on the daily temperature of consumptives in the stage of softening, and I have noticed a rise of from one to two degrees. Any observant physician, whose attention is directed to it, will not fail to perceive the disastrous effect of persistent exercise in respect to an exacerbation of all the symptoms. Whilst I thus strongly insist on the necessity for rest when there is any considerable elevation ef temperature in phthisis, I do not mean to apply this dictum to all cases. In the cases of incipient phthisis, fibroid lung, and chronic forms, with little fever, I advise an outdoor life, and exercise consistent with the stamina of the individual. Experience has abundantly shown that in cases of consumption at the initial stage, an outdoor life of some considerable exposure, indeed, is permanently beneficial. Furthermore, gentlemen, I must relieve your minds of the impression, if you have received it, that I mean by *rest*, confinement to the foul air or other evil hygienic influences of the sick room. By no means. Rest is not incompatible with constant, or nearly constant, exposure to the external air, and this I always insist upon in prescribing the necessary regimen.

I come now to another symptom—cough—which usually taxes severely the resources of the physician. As cough prevents sleep, destroys rest, and is exhausting, the patients are clamorous for relief. Much coughing is a peculiarly severe form of exercise, and demands suppression or amelioration for this reason. The expedients are almost past computation—a sure indication both of failure and intractability. The reflex irritation proceeding from the fauces may be allayed by a gargle of bromide of potassium, by brushing over the mucous membrane a one per cent. solution of carbolic acid, or by atomizing a solution of morphia. The combination of diluted hydro-bromic acid, and spirit of chloroform proposed by Dr. Fothergill, does very well sometimes, but in general is disappointing. In fact there are no efficient substitutes for opium, and we must turn to this when the cough is severe and persistent. The most generally useful of the preparations and derivatives of opium is codeia. The grounds of its utility in cough, are these : it has a selective action on the pneumogastric nerve, allaying irritability of its end organs; it is calmative and hypnotic, and as compared to morphia is less excitant and less nauseant. Codeia is adapted to those cases in which the cough is largely nervous. It may be combined with strychnia when there is vomiting, and with atropia or picro-toxine when the sweats are profuse. The following are examples: ℞. Codeiæ sulph. gr. x. ext. hyoscyami ℈j. M. ft. pil. no. xx. Sig. One pill every four hours. ℞. Codeiæ sulph. gr. xvj. strychniæ sulph, gr. j, atropiæ sulph, gr. $\frac{1}{8}$, acid, sulph. dil. ʒ ij, aquæ ʒ vj. M. Sig. Ten to fifteen drops three times a day. Morphia may be substituted for codeia in any of these prescriptions, by reducing the quantity one-half. Carbolic acid exercises no little influence over cough, expectoration, and fever, and is most serviceable in allaying the reflex vomiting. It is best given in solution, as follows: ℞. Acid. carbolic, gr. viij, aquæ laurocerasi, aquæ āā ℥ i. M. Sig. A teaspoonful every four hours. Carbolic acid is especially indicated in the fetid expectoration of bronchiectasis. In the cough of fibroid lung, and of the stage of deposit before softening in caseous pneumonia, I have had excellent results from the administration of iodide of ammonium in the wine of tar. ℞. Ammonii iodid. ʒ ij, vini picis liquid; syrp. tolu āā ℥ ij. M. Sig. A teaspoonful.

Now, gentlemen, I come to the last topic which I can discuss to-day—night sweats. This is both an interesting and an important subject. It is interesting because of the striking results obtained by some new contributions to our therapeutical resources, and important because of the baleful influence of the sweats over the progress of the disease. If the sweats are profuse, there is an actual loss of material, of salts and organic matter, which represents waste of tissue. It is highly important to check this waste and preserve the material for the nutrition of the body. Until the recent observations of Dr. Murrell proving the great value of picrotoxine, atropia had the first place as a remedy for the night sweats of phthisis. I have usually given atropia with strychnia and morphia for the triple object of arresting the sweating, allaying cough, and stopping the reflex vomiting. It is not a little remarkable that the temperature is generally reduced by the combined use of these remedies. I have preferred to give the atropia with regularity three times a day, because of an influence over the progress of the disease, which seems to be independent of its anhydrotic power, and which can be explained only on the supposition that it has the power to improve the nutrition of the lungs. Its introduction into use as a remedy for phthisis has put a new phase on the prognosis of cases of caseous pneumonia, not advanced to the stage of softening. Some practitioners prefer to give atropia in a single full dose (1.60th of a grain) at bed hour, but the results are better, if given in a small quantity (1.200th of a grain) the effect being distributed through the day. The susceptibility to the action of atropia varies greatly, and as immense discomfort may be produced by a medicinal dose, care is necessary to avoid unpleasant results. Recognizing the importance of the influence which atropia appears to have on the trophic system of the lungs, I have had patients take it for years at a time, and without any ill effects.

The success of picrotoxine as an anhydrotic has been quite decided. Dr. Murrell finds that so small a quantity as the 1.200th of a grain, given at bed hour, may arrest the sweating for a number of days. Although possessed of properties somewhat like those of strychnia, it is by no means so powerful, and may be given by the stomach up to 1.30th of a grain. Dover's powder, and, oddly enough, pilocarpine occasionally, acts very decidedly as an arrester of perspiration—a capital illustration of certain kinds of physiological antagonism. But pilocarpine cannot be depended on for constant service. Take it all in all, atropia should be preferred in most cases, because of its apparent influence over the nutrition of the lungs. Whilst atropia is given, three times a day, picrotoxine may be exhibited at bedtime, if the effect of the former is inadequate.

There are other important topics connected with the treatment of phthisis which I would gladly enter on, if there were sufficient time, but in the facts I have laid before you will be found the main points from which the details may be easily derived. You will find, indeed, that treated according to the principles I have just developed, many cases of phthisis will be conducted ultimately to a safe termination, and you will learn to look with more hopeful confidence on the success of your management than you have probably done heretofore.—*Medical News and Abstract.*

---

IN response to the challenge for the production from the experience of our American surgeons results which demonstrate any superiority in the methods of Mr. Lister over those less complicated and more frequently adopted, and to meet also the demand that we should compare the results of the different methods adopted by surgeons of to-day, I want to call attention to the labors of my friend, Professor Gerrish, of the Maine General Hospital, who last spring published the results of such a comparison made in the wards of that hospital. He was an enthusiastic adherent of antiseptic surgery according to Lister; among his colleagues was Dr. Wm. Warren Green, referred to in one of the papers of the evening. Professor Green was not an adherent of Lister. Professor Gerrish treated six (6) cases of excision of the breast according to Listerian methods. His colleagues excised the breast in seven (7) cases. They were all in the same ward, and cared for by the same nurses, fed with the same food, and their conditions were as near as possible the same. It is not to be forgotten that their constitutional differences are to be looked after, and it was stated that the constitutional conditions of those treated by ordinary methods was quite as good, if not better, than those treated antiseptically. The results were as follows: The average number of days in hospital of patients treated antiseptically, was 12$\frac{2}{3}$. When they left the hospital, every one left with the wound entirely closed, except in one case. Of those who were treated according to the ordinary methods, the average stay in hospital was 23$\frac{4}{7}$ days, and in only three cases had complete healing of the wound taken place at the time of the discharge. Of the cases treated antiseptically, in every one was union by first intention obtained without suppuration, except in one case, in which atmospheric air was accidentally allowed access to the wound. Of the non-antiseptic cases, in all there was suppuration. Here, then, has been performed a practical experiment, by one of our own colleagues, in conditions that were especially favorable for rendering the results decisive, a real *experimentum crucis.* The results need no comment.—*Pilcher.*

