# Correction

**Published:** 1882-02

**Authors:** 


CORRECTION.—The article on Injuries to the Head, which appeared on page 65 of the January number of the JOURNAL, was erroneously credited to the Annals of the New York Surgical Society. It should have read ANNALS OF ANATOMY AND SURGERY. We have had frequent occasion to make extracts from the pages of this valuable periodical; and take occasion to say, that under the management of its distinguished editor, Dr. James E. Pilcher, it constitutes a storehouse of science which should be in the hands of every practitioner of surgery.

ACTION OF DUBOISIA ON THE CIRCULATION.—Dr. Gibson has a memoir on this subject in the *Journal of Anatomy and Physiology.* The properties of duboisia have been investigated by Mr. Tweedy, Dr. Ringer and Dr. Fraser, and they have shown that it dilates the pupil, dries the mouth, quickens the pulse, arrests perspiration, produces headache, causes drowsiness, and finally induces tetanus. The conclusions at which Dr. Gibson has arrived are: 1. That duboisia in quantities not exceeding 0.005 gram, raises the arterial blood-pressure without materially affecting the pulse-rate. 2. In quantities not exceeding 0.05 gram it diminishes the blood-pressure and lessens the pulse-rate. 3. In quantities of 0.05 gram and upward it causes death, with the heart in a state of diastole. 4. Upon the heart itself duboisia has but little action, except in very large doses—*i. e.*, doses of more than 0.05 gram—and it then causes arrest of the heart in diastole. 5. Duboisia stimulates the central inhibitory mechanism. 6. The alkaloid paralyzes the peripheral inhibitory apparatus. 7. Duboisia stimulates the central vaso-motor apparatus and causes contraction of the arterioles in small doses; in large doses it lowers the activity of the central vaso-motor mechanism, and dilates the arterioles. 8. Duboisia has no influence over the sympathetic nerve.—*Louisville Medical News.*

